# Generation of spin currents by surface plasmon resonance

**DOI:** 10.1038/ncomms6910

**Published:** 2015-01-08

**Authors:** K. Uchida, H. Adachi, D. Kikuchi, S. Ito, Z. Qiu, S. Maekawa, E. Saitoh

**Affiliations:** 1 Institute for Materials Research, Tohoku University, Sendai 980-8577, Japan; 2 PRESTO, Japan Science and Technology Agency, Saitama 332-0012, Japan; 3 Advanced Science Research Center, Japan Atomic Energy Agency, Tokai 319-1195, Japan; 4 CREST, Japan Science and Technology Agency, Tokyo 102-0075, Japan; 5 WPI Advanced Institute for Materials Research, Tohoku University, Sendai 980-8577, Japan

## Abstract

Surface plasmons, free-electron collective oscillations in metallic nanostructures, provide abundant routes to manipulate light–electron interactions that can localize light energy and alter electromagnetic field distributions at subwavelength scales. The research field of plasmonics thus integrates nano-photonics with electronics. In contrast, electronics is also entering a new era of spintronics, where spin currents play a central role in driving devices. However, plasmonics and spin-current physics have so far been developed independently. Here we report the generation of spin currents by surface plasmon resonance. Using Au nanoparticles embedded in Pt/BiY_2_Fe_5_O_12_ bilayer films, we show that, when the Au nanoparticles fulfill the surface-plasmon-resonance conditions, spin currents are generated across the Pt/BiY_2_Fe_5_O_12_ interface. This spin-current generation cannot be explained by conventional heating effects, requiring us to introduce nonequilibrium magnons excited by surface-plasmon-induced evanescent electromagnetic fields in BiY_2_Fe_5_O_12_. This plasmonic spin pumping integrates surface plasmons with spin-current physics, opening the door to plasmonic spintronics.

## Abstract (web summary)

Optical methods allow for the excitation of diverse magnetic phenomena in nanostructured materials. Here, Uchida *et al*. demonstrate how pure spin current may be generated across a Pt/BiY_2_Fe_5_O_12_ thin film interface by optically exciting surface plasmon resonance in embedded gold nanoparticles.

## Graphical abstract

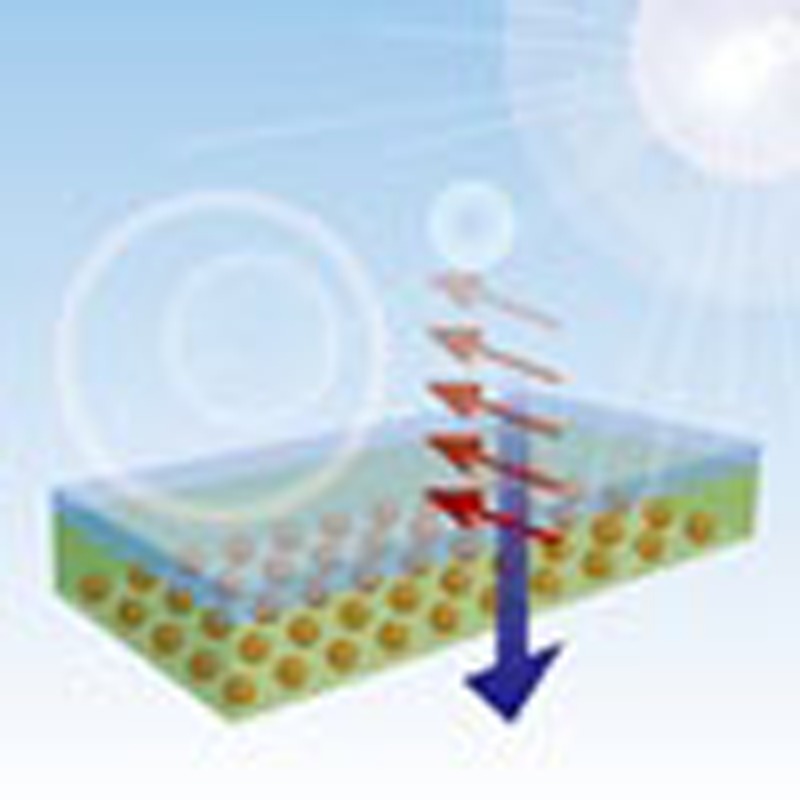

Surface plasmons enable subdiffraction-limit localization of light and strong enhancement of electromagnetic fields. Owing to this feature, plasmonics^1^,^2^,^3^,^4^,^5^ has vast potential in solar cells, light generation, microscopy, data storage and bio-sensors. One of the other emerging developments in this field is magneto-plasmonics^6^,^7^, which offers unique possibilities to manipulate light by the use of external magnetic fields or magnetic materials. Important topics in magneto-plasmonics include the enhancement of magneto-optical effects in plasmonic nanostructures^6^, influence of magnetic fields on surface plasmon resonance (SPR) and observation of SPR in ferromagnetic metals^7^. However, the integration of plasmonics into spintronics^8^,^9^ is yet to be realized, and the interaction between surface plasmons and spin currents^10^,^11^ contains unexplored physics.

In the field of spintronics, for spin-current generation, we often rely on a spin pumping effect^12^,^13^,^14^,^15^, which refers to the transfer of spin-angular momentum from magnetization dynamics in a ferromagnet to conduction-electron spins in an attached paramagnet; when a motion of magnetic moments in the ferromagnet is excited, a spin current is pumped out of the ferromagnet into the paramagnet.

Recent studies in spintronics have revealed that there are two types of spin pumping effects. One is coherent spin pumping, which is due to coherent phase precession of the magnetization, that is, ferromagnetic or spin-wave resonances in ferromagnets typically excited by microwave irradiation^13^,^14^,^15^. The other is incoherent spin pumping, which is due to a nonequilibrium state of spins at a ferromagnet/paramagnet interface excited by incoherent external perturbations; when magnons in the ferromagnet and/or electrons in the paramagnet deviate from thermal equilibrium, a net spin current is generated across the interface. A typical example of the incoherent spin pumping is the spin Seebeck effect^16^,^17^,^18^,^19^,^20^,^21^,^22^,^23^,^24^, where the magnon/electron distribution functions are modulated by external temperature gradients.

In this work, we report the observation of plasmonic spin pumping; we show that the incoherent spin pumping can be driven by surface plasmons under visible light illumination. This plasmonic spin pumping will invigorate research in both the fields of spintronics and plasmonics, paving the way to create new devices, such as solar spin-current generators and spin-based plasmonic sensors.

## Results

### Device structure

**Figure 1:**
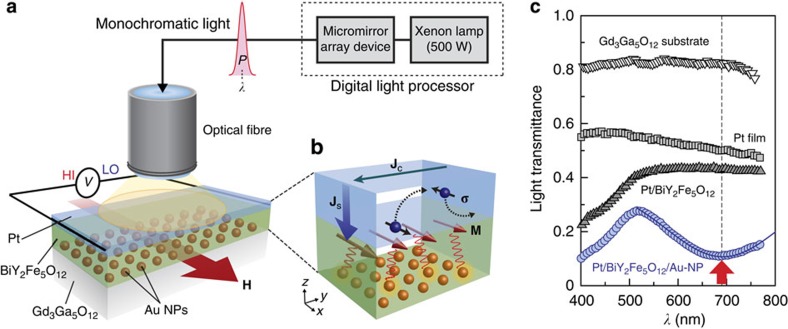
Experimental set-up and device structure. (**a**) A schematic illustration of the Pt/BiY_2_Fe_5_O_12_/Au-nanoparticle (NP) sample and experimental configuration for measuring the plasmonic spin pumping. The Pt/BiY_2_Fe_5_O_12_/Au-NP sample is of a 10 mm × 5 mm rectangular shape. The sample was illuminated with monochromatic light with the wavelength *λ* (400–770 nm), power *P*, bandwidth of 20 nm and spot diameter of ~5 mm from the Pt-film side at normal incidence by using a digital light processor comprising a 500 W Xenon lamp and a micromirror array device (Gooch & Housego, OL490). HI and LO represent the inputs of a voltmeter. **H** denotes the external magnetic field vector (red arrow) with the magnitude *H*. (**b**) Plasmonic spin pumping and inverse spin Hall effect (ISHE) in the Pt/BiY_2_Fe_5_O_12_/Au-NP sample. Here, **M**, **J**_C_, **J**_S_ and **σ** denote the magnetization vector (dark red arrows), charge current generated by the ISHE (green arrow), spatial direction of the spin current generated by the plasmonic spin pumping (blue arrow) and spin-polarization vector of the spin current (black arrows), respectively. Blue (orange) spheres schematically show electron charge (Au NPs). Black dotted arrows and red wavy lines schematically show the trajectory of the electrons and the interaction between magnetic moments in the BiY_2_Fe_5_O_12_ layer and evanescent electromagnetic fields induced by the surface plasmon resonance (SPR), respectively. (**c**) *λ* dependence of the light transmittance of the 0.5-mm-thick Gd_3_Ga_5_O_12_ substrate, 5-nm-thick Pt film on the Gd_3_Ga_5_O_12_ substrate, Pt/BiY_2_Fe_5_O_12_ sample and Pt/BiY_2_Fe_5_O_12_/Au-NP sample. In the Pt/BiY_2_Fe_5_O_12_ sample, the Pt and BiY_2_Fe_5_O_12_ films were formed directly on the Gd_3_Ga_5_O_12_ substrate without Au NPs. A blue solid curve was obtained by fitting the observed dip structure using a Gaussian function. A red arrow shows the wavelength at which the SPR is excited in the Pt/BiY_2_Fe_5_O_12_/Au-NP sample.

**Figure 2:**
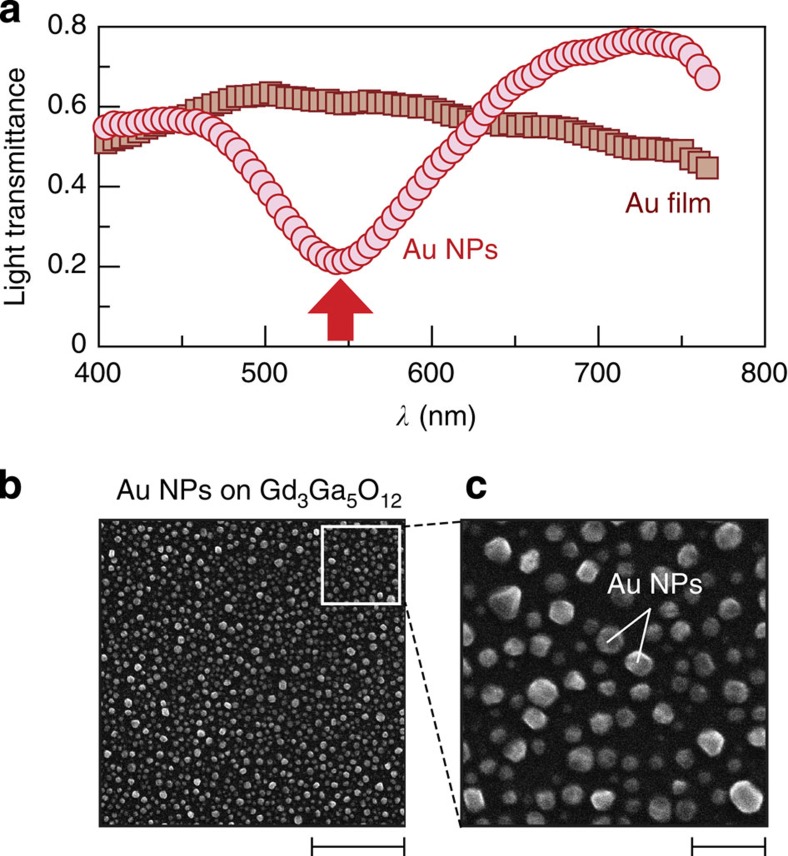
Au nanoparticles. (**a**) *λ* dependence of the light transmittance of a 15-nm-thick Au film and Au NPs on the Gd_3_Ga_5_O_12_ substrate. The light-transmittance spectrum of the Au-NP sample exhibits a clear dip structure due to the SPR, while that of the Au film does not exhibit strong *λ* dependence. The position of the SPR wavelength for the Au-NP sample is marked with a red arrow. (**b**) Scanning electron microscope image of Au NPs on the Gd_3_Ga_5_O_12_ substrate, measured before forming the BiY_2_Fe_5_O_12_ and Pt layers. (**c**) Magnified scanning electron microscope image of the Au NPs on the Gd_3_Ga_5_O_12_ substrate. The scale bars in (**b**) and (**c**) represent 1 μm and 200 nm, respectively.

Figure 1a shows a schematic illustration of the sample system used in the present study. The sample consists of a paramagnetic Pt/ferrimagnetic BiY_2_Fe_5_O_12_ bilayer film with Au nanoparticles (NPs) embedded in the BiY_2_Fe_5_O_12_ layer. The Au NPs with an in-plane diameter of 30–90 nm and a height of 20–50 nm were formed by heating a Au thin film^6^ on a single-crystalline Gd_3_Ga_5_O_12_ (111) substrate (Fig. 2b,c). The 110-nm-thick BiY_2_Fe_5_O_12_ film was then coated on them by means of a metal-organic decomposition (MOD) method^22^ (see Methods). Finally, a 5-nm-thick Pt film was sputtered on to the BiY_2_Fe_5_O_12_ film. To excite surface plasmons, the Pt/BiY_2_Fe_5_O_12_/Au-NP sample was illuminated with unpolarized monochromatic light with wavelength *λ* (400–770 nm) and power *P* from the Pt-film side at normal incidence (Fig. 1a). As the Pt and BiY_2_Fe_5_O_12_ layers are very thin, the light passes through the layers and interacts with the Au NPs. We checked that the electric resistance between the ends of the BiY_2_Fe_5_O_12_ layer with Au NPs under the light illumination is much greater than the measurement limit of our electrometer (>2 × 10^11^ Ω) at all the *λ* values; the possibility of optical charge-carrier generation in BiY_2_Fe_5_O_12_ is excluded.

In Fig. 1c, we show the light-transmittance spectrum of the Pt/BiY_2_Fe_5_O_12_/Au-NP sample (see the blue circle data points). We found that the sample exhibits a clear dip structure in the transmittance spectrum around *λ*=690 nm in comparison with the spectra of a Gd_3_Ga_5_O_12_ substrate, a plain Pt film and Pt/BiY_2_Fe_5_O_12_ sample without Au NPs. This dip structure for the Pt/BiY_2_Fe_5_O_12_/Au-NP sample is attributed to the excitation of localized SPR^1^,^2^ in the Au NPs embedded in the BiY_2_Fe_5_O_12_ layer (see also Fig. 2a).

### Electromagnetic field distributions

**Figure 3:**
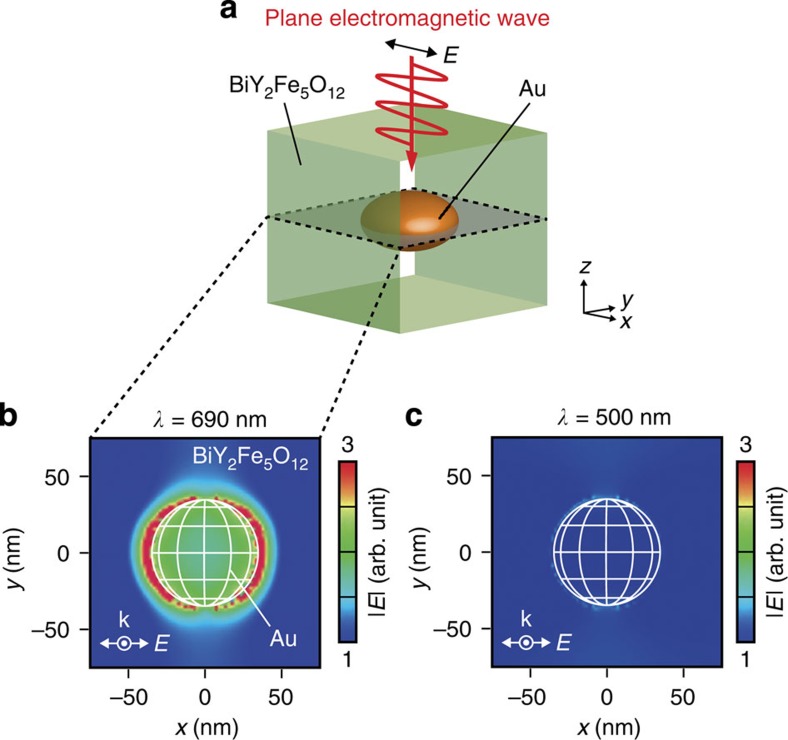
Numerical calculation. (**a**) A schematic illustration of the BiY_2_Fe_5_O_12_/Au-NP model used for the numerical calculation based on a finite-difference time-domain method. Plane electromagnetic waves with the polarization along the *x* direction and the wave vector **k** along the *z* direction are applied to the BiY_2_Fe_5_O_12_/Au-NP model. *E* denotes an electric field of the electromagnetic waves. (**b**,**c**) Simulated distributions of the electric field intensity |*E*| in the BiY_2_Fe_5_O_12_/Au-NP model in the *x*-*y* plane across the centre of the Au NP at *λ*=690 nm (**b**) and 500 nm (**c**).

To visualize the SPR in the BiY_2_Fe_5_O_12_/Au-NP structure, we performed electromagnetic field simulations by means of a finite-difference time-domain (FDTD) method^25^. Figure 3a shows a schematic illustration of a BiY_2_Fe_5_O_12_/Au-NP model used for the FDTD simulations (see Methods for details). As shown in Fig. 3b, under the SPR condition (*λ*=690 nm), strong evanescent electromagnetic fields, that is, near fields^1^,^2^, are generated in the BiY_2_Fe_5_O_12_ film in the vicinity of the BiY_2_Fe_5_O_12_/Au-NP interface, while the enhancement and localization of electromagnetic fields do not appear at *λ*=500 nm (Fig. 3c), consistent with the light-transmittance spectrum of the Pt/BiY_2_Fe_5_O_12_/Au-NP sample in Fig. 1c. The FDTD simulations also show that the evanescent electromagnetic fields in the BiY_2_Fe_5_O_12_ layer due to the SPR are not affected by the presence of the Pt layer irrespective of the position and size of Au NPs (Supplementary Figs 1 and 2).

### Measurement mechanism of plasmonic spin pumping

In the Pt/BiY_2_Fe_5_O_12_/Au-NP sample, if the evanescent electromagnetic fields concomitant with surface plasmons in Au NPs excite magnons in the BiY_2_Fe_5_O_12_ film and the excited magnons drive the incoherent spin pumping, a spin current is generated in the Pt layer with the spatial direction **J**_S_ and the spin-polarization vector **σ** parallel to the magnetization **M** of the BiY_2_Fe_5_O_12_ film (Fig. 1b). This plasmon-induced spin current is converted into a d.c. charge current **J**_C_ due to the inverse spin Hall effect (ISHE)^14^,^26^,^27^ in the Pt layer owing to the strong spin-orbit interaction in Pt. When **M** of the BiY_2_Fe_5_O_12_ film is along the *x* direction, **J**_C_ is generated along the *y* direction because of the following ISHE symmetry:


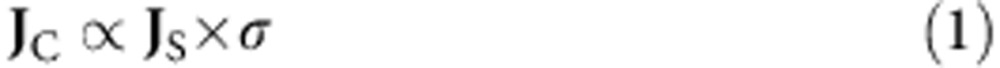


where the *x*, *y* and *z* directions are defined in Fig. 1b. We note that extrinsic artefacts induced by a static magnetic proximity effect^28^ at the Pt/BiY_2_Fe_5_O_12_ interface are negligibly small in the present structure^23^,^24^. To detect the ISHE induced by the plasmonic spin pumping, we measured an electric voltage *V* between the ends of the Pt layer under open circuit condition while illuminating with monochromatic light and applying an external magnetic field **H** (with the magnitude *H*) to the Pt/BiY_2_Fe_5_O_12_/Au-NP sample along the *x*-*y* plane (Fig. 1a). All of the *V* measurements were carried out at room temperature and atmospheric pressure.

### Observation of plasmonic spin pumping

**Figure 4:**
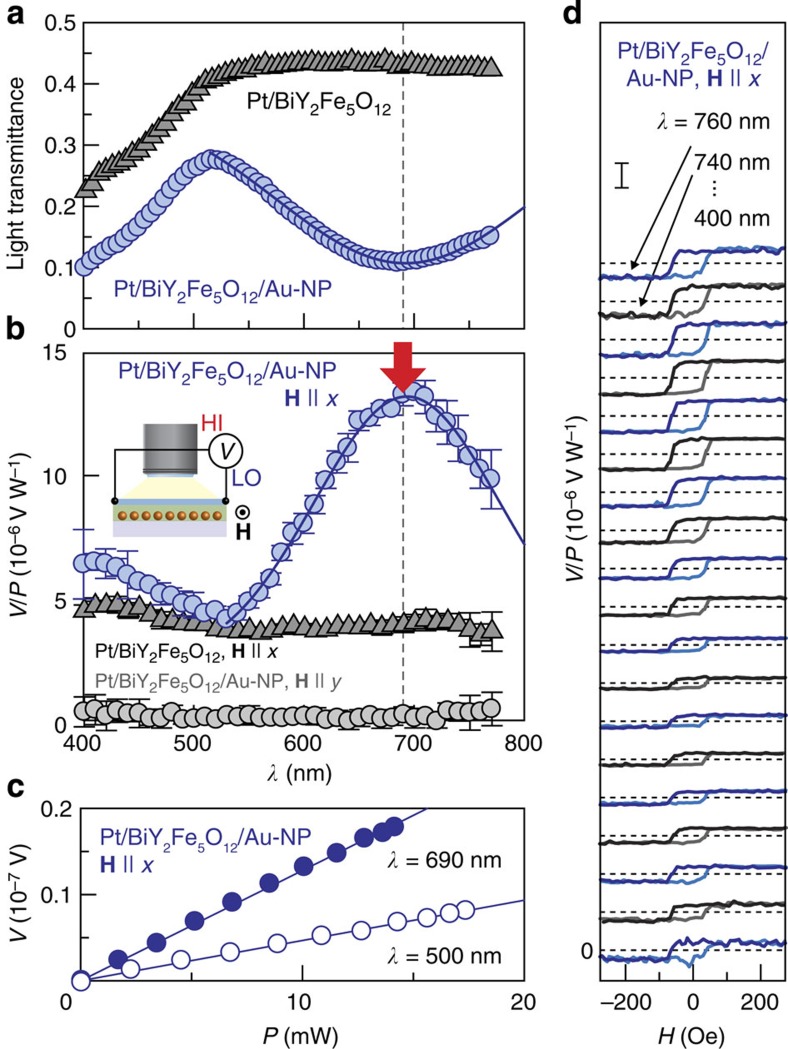
Observation of plasmonic spin pumping. (**a**) *λ* dependence of the light transmittance of the Pt/BiY_2_Fe_5_O_12_/Au-NP and Pt/BiY_2_Fe_5_O_12_ samples. The samples were prepared at the same time. (**b**) *λ* dependence of the electric voltage between the ends of the Pt layer normalized by the incident light power, *V*/*P*, in the Pt/BiY_2_Fe_5_O_12_/Au-NP and Pt/BiY_2_Fe_5_O_12_ samples, measured when **H** of *H*=200 Oe was applied along the *x* or *y* direction. The in-plane coercive force of the BiY_2_Fe_5_O_12_ layer is around 30 Oe; the magnetization of BiY_2_Fe_5_O_12_ is aligned along the **H** direction at *H*=200 Oe. The position of the SPR wavelength is marked with a red arrow. A blue solid curve was obtained by fitting the observed peak voltage structure using a Gaussian function. The error bars represent the s.d. of the measurements. (**c**) *P* dependence of *V* in the Pt/BiY_2_Fe_5_O_12_/Au-NP sample at *λ*=690 nm and 500 nm, measured when **H** of *H*=200 Oe was applied along the *x* direction. (**d**) *H* dependence of *V*/*P* in the Pt/BiY_2_Fe_5_O_12_/Au-NP sample for various values of *λ* (=760, 740,..., 400 nm), measured when **H** was along the *x* direction. The scale bar represents *V*/*P*=20 × 10^−6^ V W^−1^. The dark blue and black lines (light blue and grey lines) show the *V*/*P* signals measured when **H** was swept from positive to negative (from negative to positive).

To observe the plasmonic spin pumping, it is important to separate the plasmon-induced signals from extrinsic heating effects. First of all, to check the heating effect due to the light illumination, we measured the voltage in the Pt/BiY_2_Fe_5_O_12_ sample without Au NPs. The grey triangle data points in Fig. 4b show the *λ* dependence of the voltage normalized by the incident light power, *V*/*P*, in the Pt/BiY_2_Fe_5_O_12_ sample at *H*=200 Oe. When **H** is applied along the *x* direction, a finite voltage signal appears in the Pt layer at all the *λ* values. Because of the absence of Au NPs and surface plasmons, the signal in the Pt/BiY_2_Fe_5_O_12_ sample is attributed to the heating of the sample by the light illumination, that is, the longitudinal spin Seebeck effect^20^,^22^,^23^,^24^ (see also Supplementary Fig. 3). Here, the sign of the *V*/*P* signal shows that the temperature of the Pt layer is higher than that of the BiY_2_Fe_5_O_12_ layer under the light illumination (Supplementary Fig. 4 and Supplementary Note 1). Importantly, this heating signal in the Pt/BiY_2_Fe_5_O_12_ sample does not exhibit strong *λ* dependence (Fig. 4b).

Now we are in a position to investigate the plasmonic generation of spin currents. The blue circle data points in Fig. 4b are the *V*/*P* spectrum in the Pt/BiY_2_Fe_5_O_12_ film that contains Au NPs. Also in this Pt/BiY_2_Fe_5_O_12_/Au-NP sample, when **H** || *x*, the *V*/*P* signal appears with the same sign (Fig. 4b) and the magnitude of *V* is proportional to the light power *P* (Fig. 4c). The sign of the signal at each *λ* was observed to be reversed by reversing *H* with a hysteresis loop, indicating that the signal is affected by the **M** direction of the BiY_2_Fe_5_O_12_ layer (Fig. 4d). As also shown in Fig. 4b, we confirmed that the *V*/*P* signal disappears when **H** is applied along the *y* direction, consistent with equation (1). These results indicate that the observed *V*/*P* signal in the Pt/BiY_2_Fe_5_O_12_/Au-NP sample is due to the ISHE induced by spin currents in the Pt layer. Significantly, Fig. 4b shows that the *V*/*P* signal in the Pt/BiY_2_Fe_5_O_12_/Au-NP sample exhibits peak structure and is markedly enhanced around *λ*=690 nm. This peak position in the *V*/*P* spectrum coincides with the SPR wavelength of this Pt/BiY_2_Fe_5_O_12_/Au-NP sample (Fig. 4a,b). As both the Pt/BiY_2_Fe_5_O_12_/Au-NP and Pt/BiY_2_Fe_5_O_12_ samples are illuminated from the Pt-film side, the temperature rise of the Pt layer is almost the same for both, confirming that the heating of the Pt layer is irrelevant to the ISHE enhancement under the SPR condition in the Pt/BiY_2_Fe_5_O_12_/Au-NP sample (see Supplementary Fig. 4 and Supplementary Note 1), where we note again that the background heating signal does not exhibit strong *λ* dependence. This peak *V*/*P* structure in the Pt/BiY_2_Fe_5_O_12_/Au-NP sample also cannot be explained by the heating of Au NPs or the BiY_2_Fe_5_O_12_ layer under the SPR condition because of the different sign; if the BiY_2_Fe_5_O_12_ layer was heated by electromagnetic loss in Au NPs, the ISHE voltage should decrease under the SPR condition as the temperature gradient generated by the Au-NP heating across the Pt/BiY_2_Fe_5_O_12_ interface is of an opposite sign to the case when the Pt layer is heated (see Supplementary Fig. 4 and Supplementary Note 1). A possible slight heating of Au NPs in the off-resonance region might reduce the magnitude of the background signal slightly, but is also irrelevant to the *V*/*P* signal with the positive peak structure. Our numerical calculations of electromagnetic field distributions based on the FDTD method show that the electromagnetic coupling between the Pt film and Au NPs under the SPR condition is too weak to explain the enhancement of the ISHE signal in the Pt/BiY_2_Fe_5_O_12_/Au-NP sample; as no SPR mode due to the coupling appears in the configuration used in this study, the temperature rise of the Pt layer is not changed by the SPR in Au NPs (Supplementary Figs 1 and 2).

**Figure 5:**
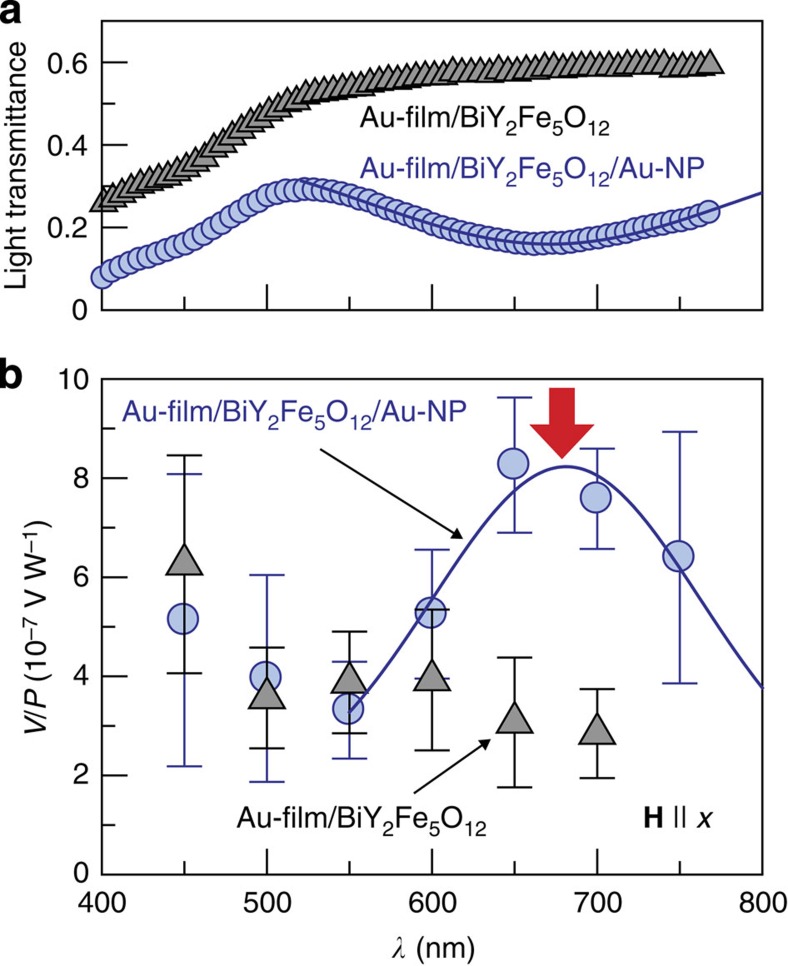
Measurements using Au film. (**a**) *λ* dependence of the light transmittance of the Au-film/BiY_2_Fe_5_O_12_/Au-NP and Au-film/BiY_2_Fe_5_O_12_ samples. (**b**) *λ* dependence of *V*/*P* in the Au-film/BiY_2_Fe_5_O_12_/Au-NP and Au-film/BiY_2_Fe_5_O_12_ samples, measured when **H** of *H*=200 Oe was applied along the *x* direction. The Au-film/BiY_2_Fe_5_O_12_/Au-NP sample was illuminated with monochromatic light with the wavelength *λ*, power *P*, bandwidth of 50 nm and spot diameter of ~5 mm from the Au-film side at normal incidence.

**Figure 6:**
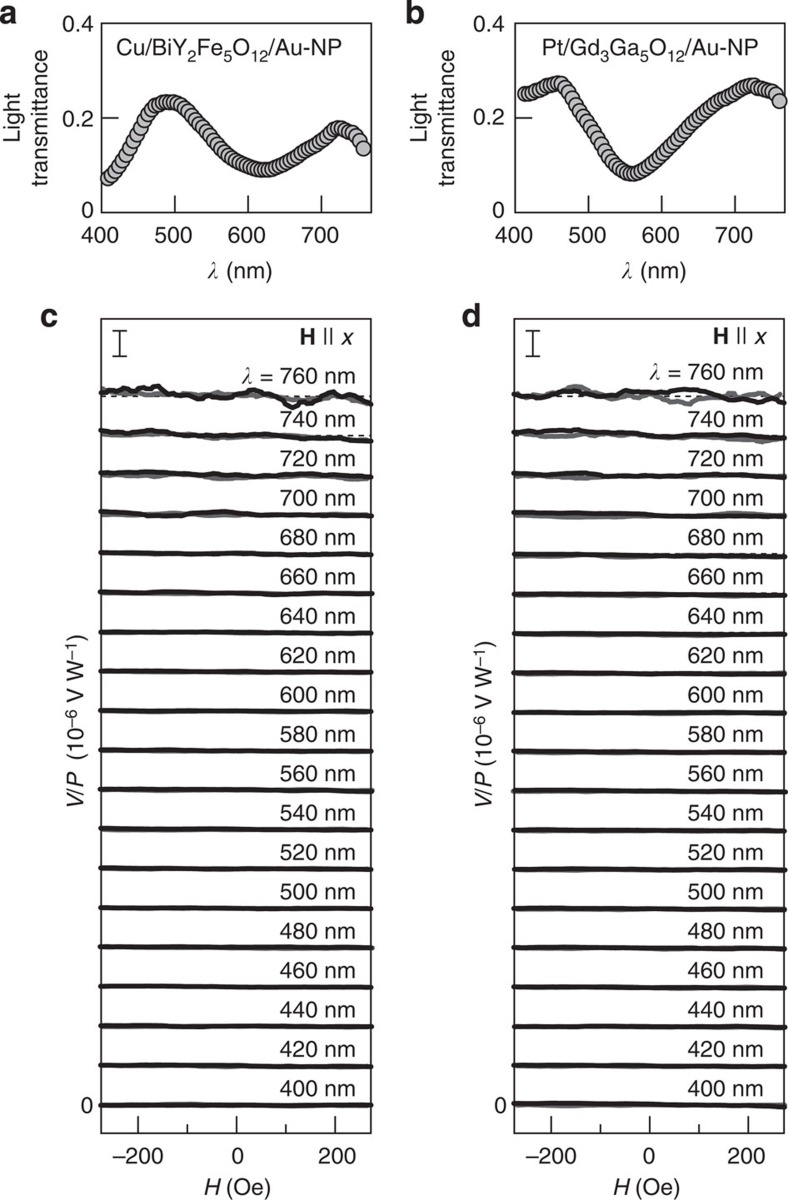
Control experiments using different materials. (**a**,**b**) *λ* dependence of the light transmittance of the Cu/BiY_2_Fe_5_O_12_/Au-NP (**a**) and Pt/Gd_3_Ga_5_O_12_/Au-NP (**b**) samples. (**c**,**d**) *H* dependence of *V*/*P* in the Cu/BiY_2_Fe_5_O_12_/Au-NP (**c**) and Pt/Gd_3_Ga_5_O_12_/Au-NP (**d**) samples for various values of *λ* (=760, 740,..., 400 nm), measured when **H** was along the *x* direction. The scale bars represent *V*/*P*=20 × 10^−6^ V W^−1^. All the control experiments were performed by using the samples of a 10 mm × 5 mm rectangular shape.

We also performed control experiments by using different materials. We found that similar *V*/*P* signals appear in a Au-film/BiY_2_Fe_5_O_12_/Au-NP sample, where the Pt film is replaced with a 5-nm-thick Au film (Fig. 5a,b); as Au is a typical metal far from ferromagnetism^29^,^30^, proximity ferromagnetism in Pt cannot be the origin of the observed peak voltage structure. In contrast, this voltage signal was found to disappear in a Cu/BiY_2_Fe_5_O_12_/Au-NP sample, where the Pt layer is replaced with a 5-nm-thick Cu film in which the spin-orbit interaction is very weak, indicating the important role of spin-orbit interaction, or the ISHE, in the voltage generation (Fig. 6a,c). The voltage signal also disappears in a Pt/Gd_3_Ga_5_O_12_/Au-NP sample, where Au NPs are embedded in a paramagnetic insulator Gd_3_Ga_5_O_12_ film instead of BiY_2_Fe_5_O_12_ (see Fig. 6b,d and Methods), indicating that direct contact between BiY_2_Fe_5_O_12_ and Pt is necessary for the observed voltage generation; electromagnetic artefacts are irrelevant. All the control experiments support our interpretation that the enhancement of the ISHE signal under the SPR condition in the Pt/BiY_2_Fe_5_O_12_/Au-NP sample results from the plasmonic spin pumping.

### Separation of plasmonic spin pumping from heating effects

**Figure 7:**
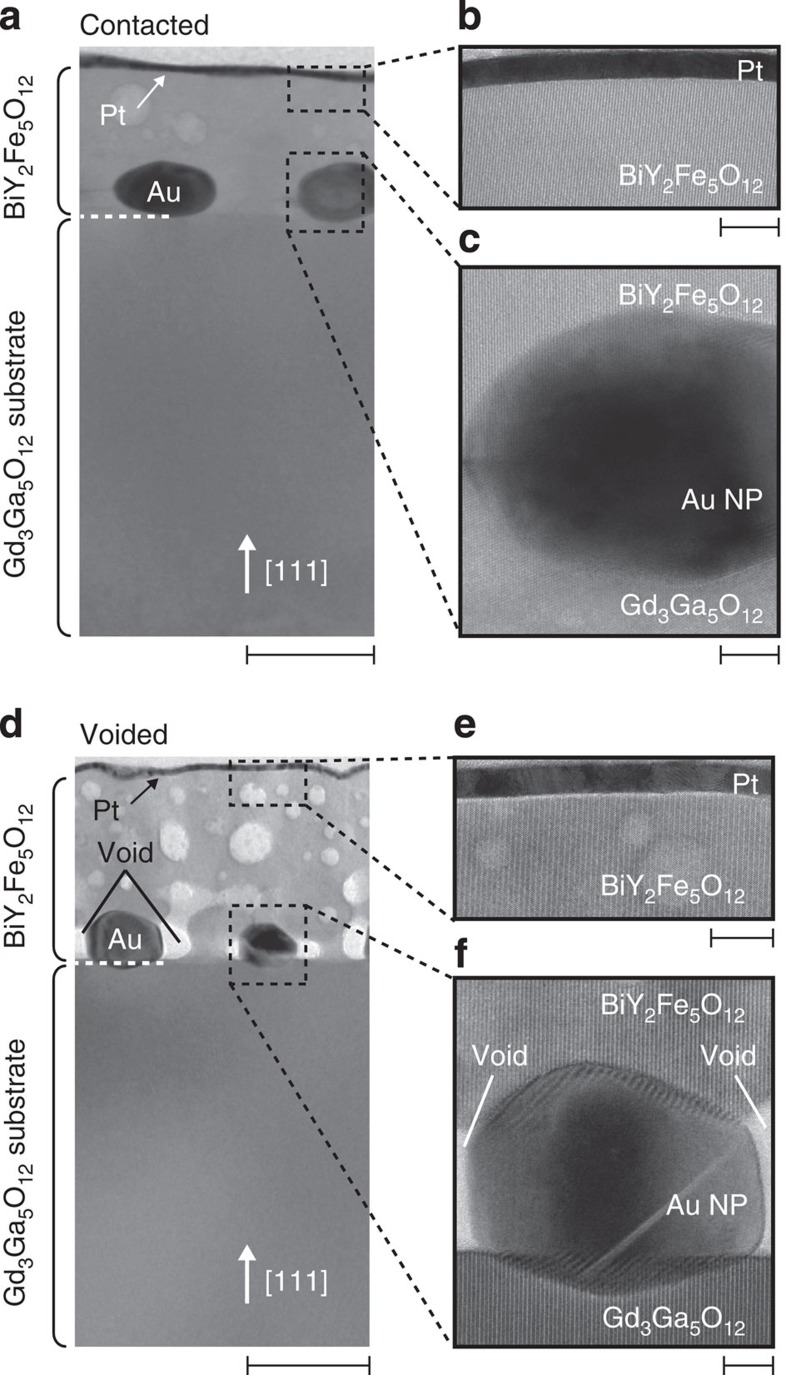
Cross-sectional images. (**a**) Cross-sectional transmission electron microscope (TEM) images of the contacted Pt/BiY_2_Fe_5_O_12_/Au-NP sample. (**b**,**c**) Magnified TEM images of the contacted Pt/BiY_2_Fe_5_O_12_/Au-NP sample near the Pt film (**b**) and the Au NP (**c**). (**d**) Cross-sectional TEM images of the voided Pt/BiY_2_Fe_5_O_12_/Au-NP sample. (**e**,**f**) Magnified TEM images of the voided Pt/BiY_2_Fe_5_O_12_/Au-NP sample near the Pt film (**e**) and the Au NP (**f**). Scale bars in (**a**) and (**d**) (**b**,**c**,**e** and **f**) represent 100 nm (10 nm). White arrows in (**a**) and (**d**) represent the crystal orientation of the Gd_3_Ga_5_O_12_ substrate. The voided Pt/BiY_2_Fe_5_O_12_/Au-NP sample has small voids with the size of 20–30 nm between the BiY_2_Fe_5_O_12_ film and Au NPs, while Au NPs in the contacted sample are densely embedded in BiY_2_Fe_5_O_12_.

**Figure 8:**
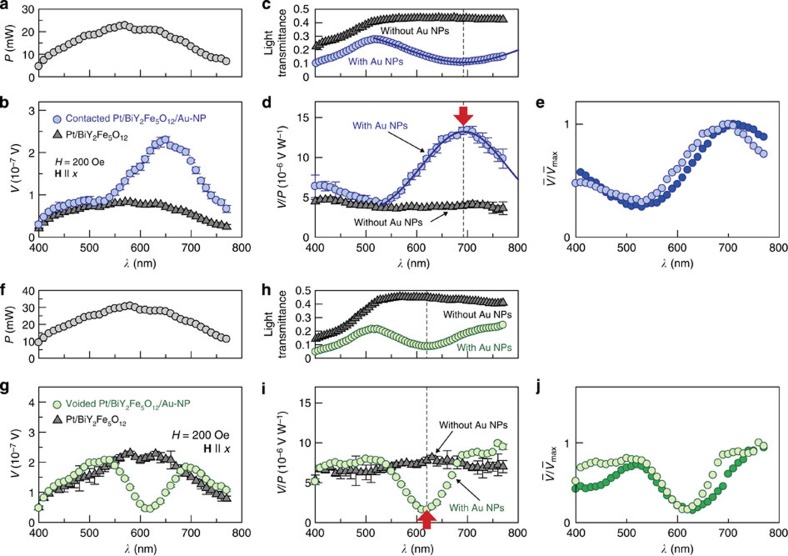
Evidence for magnon excitation by surface plasmons. (**a**) *λ* dependence of *P*, applied when the data in (**b**) were measured. (**b**) *λ* dependence of *V* in the contacted Pt/BiY_2_Fe_5_O_12_/Au-NP and Pt/BiY_2_Fe_5_O_12_ samples, measured when **H** of *H*=200 Oe was applied along the *x* direction. The error bars represent the s.d. of the measurements. (**c**) *λ* dependence of the light transmittance of the contacted Pt/BiY_2_Fe_5_O_12_/Au-NP and Pt/BiY_2_Fe_5_O_12_ samples. (**d**) *λ* dependence of *V*/*P* in the contacted Pt/BiY_2_Fe_5_O_12_/Au-NP and Pt/BiY_2_Fe_5_O_12_ samples. (**e**) *λ* dependence of 
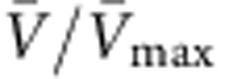
 in two different contacted Pt/BiY_2_Fe_5_O_12_/Au-NP samples, where 
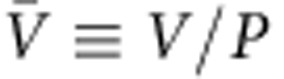
 and 
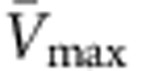
 is the maximum value of 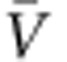 in the 
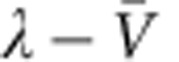
 spectrum. (**f**–**j**) Experimental results for the voided Pt/BiY_2_Fe_5_O_12_/Au-NP and Pt/BiY_2_Fe_5_O_12_ samples. (**j**) shows the *λ* dependence of 
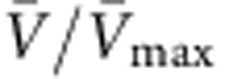
 in two different voided Pt/BiY_2_Fe_5_O_12_/Au-NP samples.

To further verify the effect of surface plasmons on the spin-current generation, we measured the *V*/*P* spectra in the Pt/BiY_2_Fe_5_O_12_/Au-NP samples on changing the contact condition between BiY_2_Fe_5_O_12_ and Au NPs. Here, we prepared the Pt/BiY_2_Fe_5_O_12_/Au-NP samples by means of two different annealing conditions for the BiY_2_Fe_5_O_12_ layer (see Methods for details). In the Pt/BiY_2_Fe_5_O_12_/Au-NP sample used for the experiments in Fig. 4, Au NPs are densely embedded in the BiY_2_Fe_5_O_12_ film (Fig. 7a–c), where the peak *V*/*P* structure appears under the SPR condition (Fig. 8a–d). In contrast, we also fabricate the Pt/BiY_2_Fe_5_O_12_/Au-NP sample in which the Au NPs are separated from the BiY_2_Fe_5_O_12_ film by small voids (Fig. 7d-f), which interrupt the interaction between magnons in BiY_2_Fe_5_O_12_ and surface plasmons in Au NPs as the evanescent electromagnetic fields generated by the SPR are localized in the vicinity of Au NPs (Supplementary Fig. 5). The difference in the SPR wavelength between the ‘contacted’ and ‘voided’ Pt/BiY_2_Fe_5_O_12_/Au-NP samples is attributed to the difference in the dielectric constant of the medium around Au NPs; As Au NPs in the voided Pt/BiY_2_Fe_5_O_12_/Au-NP sample are surrounded by voids, its SPR wavelength is closer to that of plain Au NPs without BiY_2_Fe_5_O_12_ (compare Figs 2a and 8c,h). In the voided Pt/BiY_2_Fe_5_O_12_/Au-NP sample, dip structure was found to appear in the *V*/*P* spectrum under the SPR condition (Fig. 8f–i). This behaviour was observed not only in one sample but also in our different samples as exemplified in Fig. 8e,j. The dip *V*/*P* signal in the voided Pt/BiY_2_Fe_5_O_12_/Au-NP sample is attributed to the heating of Au NPs by the SPR, which generates the temperature gradient across the Pt/BiY_2_Fe_5_O_12_ interface and the longitudinal spin Seebeck voltage along the direction opposite to the case when the Pt layer is heated (see Supplementary Figs 4,5 and Supplementary Note 1). These results indicate that the enhancement of the ISHE under the SPR condition, observed in the contacted Pt/BiY_2_Fe_5_O_12_/Au-NP sample, requires direct contact between the BiY_2_Fe_5_O_12_ and the Au NPs and cannot be explained by conventional heating effects, providing evidence for the operation of the plasmonic spin pumping due to nonequilibrium magnons excited by surface plasmons.

## Discussion

The above experiments clearly show that the spin current generated by the light illumination in the contacted Pt/BiY_2_Fe_5_O_12_/Au-NP sample is strongly enhanced under the SPR condition, and this enhancement is not connected to extrinsic heating effects. So then, what is the origin of the spin current under the SPR condition? Following previous studies on the incoherent spin pumping^31^,^32^, to generate the positive ISHE voltage in the present configuration, magnons in the BiY_2_Fe_5_O_12_ layer should be excited (see Supplementary Notes 1 and 2). A potential candidate for the magnon-excitation mechanism in the present sample structure is the strong evanescent electromagnetic fields (near-field photons) concomitant with surface plasmons. If the near-field photons excite magnons via the magnon–photon interaction^33^,^34^,^35^,^36^ near the BiY_2_Fe_5_O_12_/Au-NP interface, the excited magnons in the BiY_2_Fe_5_O_12_ layer produce a spin current owing to the incoherent spin-pumping mechanism and the positive ISHE voltage in the Pt layer: this is the plasmonic spin pumping. Using a standard many-body technique, we formulate theoretically the plasmonic spin pumping based on magnon-photon Raman scattering process (Supplementary Fig. 6). The spin current pumped by light illumination is described as


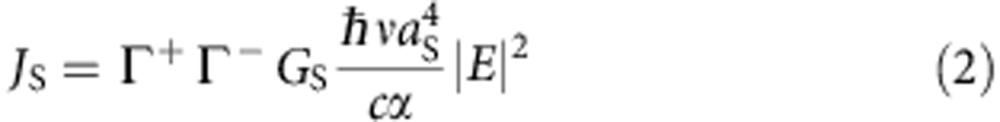


within the linear response region (see Supplementary Note 2 for details). Here, Γ^+(−)^ is the dimensionless magnon–photon coupling constant, *G*_S_ the strength of the magnetic coupling at the Pt/BiY_2_Fe_5_O_12_ interface, *ħ* the Planck constant divided by 2π, *v* the photon frequency, *c* the velocity of light, 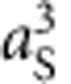 the effective block spin volume, *α* the Gilbert damping constant and *E* the electric field amplitude induced by surface plasmons. Equation (2) indicates that the plasmon-induced spin current in the Pt/BiY_2_Fe_5_O_12_/Au-NP sample is proportional to the electromagnetic energy in the BiY_2_Fe_5_O_12_ layer (∝|*E*|^2^), a situation consistent with the experimental results in Fig. 4c as |*E*|^2^ generated by surface plasmons is proportional to the incident light power *P*. The observed peak ISHE voltage in the contacted Pt/BiY_2_Fe_5_O_12_/Au-NP sample is attributed to the strong evanescent electromagnetic fields enhanced by surface plasmons, while the spin current described by Equation (2) is undetectably small in the plain Pt/BiY_2_Fe_5_O_12_ film due to the small energy transfer from external photons to magnons (see Supplementary Note 2). Note that the ratio of the voltage enhancement in Fig. 4b does not correspond to that of the |*E*|^2^ enhancement due to the presence of the background heating signal.

The plasmonic spin pumping observed here can be applied to the construction of light/spin-current convertors for driving spintronic devices. The plasmonic spin pumping also adds a light/voltage conversion function to spin Seebeck thermoelectric devices^22^, enabling hybrid voltage generation from both light and heat in a single device. The plasmonic spin pumping follows the same scaling law as the spin Seebeck effect: the output power is increased simply by extending the device area. This light/voltage conversion is conceptually different from that based on a PN junction, and is free from the output-voltage limitation caused by built-in potential. Although the spin and electric voltages generated by the plasmonic spin pumping are still small, there is plenty of scope for performance improvement; they can be enhanced by using magnetic insulators with large magnon–photon coupling, improving magnetic-insulator/NP interfaces for efficient magnon-plasmon energy transfer, and introducing plasmonic crystals that exhibit sharp and strong SPR. Plasmonic spintronics based on the interaction between surface plasmons and spin currents is still in its infancy; more detailed experimental and theoretical investigations are necessary to obtain full understanding of the mechanism of the plasmonic spin pumping and to quantitatively evaluate plasmon-magnon-electron conversion efficiency, which might be realized by removing the size, position and shape variations of Au NPs.

## Methods

### Preparation process of Pt/BiY_2_Fe_5_O_12_/Au-NP samples

The Au NPs were formed by the following two-step procedures: fabrication of a 5-nm-thick Au thin film on a Gd_3_Ga_5_O_12_ (111) substrate by using a d.c. sputtering system and heating of the Au film at 1,000 °C for 30 min (ref. 6). To increase the density of Au NPs, these procedures were repeated three times. The BiY_2_Fe_5_O_12_ thin film was then formed by the MOD method^22^. The MOD solution for the contacted (voided) Pt/BiY_2_Fe_5_O_12_/Au-NP sample includes Bi, Y and Fe carboxylates, dissolved in organic solvents with the concentration of 3% (5%). Its chemical composition is Bi:Y:Fe=1:2:5. The solution for the contacted (voided) sample was spin-coated on the Gd_3_Ga_5_O_12_ substrate with Au NPs at 5,000 r.p.m. for 60 s, followed by a drying step at 50 °C (170 °C) for 3 min and pre-annealing at 480 °C (550 °C) for 1 h (5 min). To increase the thickness of the BiY_2_Fe_5_O_12_ film, the processes from spin-coating to pre-annealing were repeated three times. Then, the contacted (voided) sample was annealed at 725 °C (680 °C) for 15 h in air to form a crystallized BiY_2_Fe_5_O_12_ film. The cross-sectional transmission electron microscope images in Fig. 7 indicate that a crystalline BiY_2_Fe_5_O_12_ film was formed on the Gd_3_Ga_5_O_12_ substrate. Finally, a 5-nm-thick Pt film was deposited over the whole surface of the BiY_2_Fe_5_O_12_ film by using an rf magnetron sputtering.

### Preparation process of Pt/Gd_3_Ga_5_O_12_/Au-NP sample

The Pt/Gd_3_Ga_5_O_12_/Au-NP sample used in Fig. 6b,d consists of a Pt/Gd_3_Ga_5_O_12_ bilayer film with Au NPs embedded in the Gd_3_Ga_5_O_12_ layer. After forming Au NPs on a Gd_3_Ga_5_O_12_ (111) substrate with the procedures described above, an additional Gd_3_Ga_5_O_12_ thin film was coated on them by means of the MOD method. The MOD solution for the Gd_3_Ga_5_O_12_ film includes Gd and Ga carboxylates, dissolved in organic solvents with the concentration of 3%. Its chemical composition is Gd:Ga=3:5. The spin-coating and annealing processes for the Gd_3_Ga_5_O_12_ film are the same as those for the BiY_2_Fe_5_O_12_ film in the contacted Pt/BiY_2_Fe_5_O_12_/Au-NP sample. Finally, a 5-nm-thick Pt film was deposited over the whole surface of the Gd_3_Ga_5_O_12_ film. The mean distance between the Pt film and Au NPs in the Pt/Gd_3_Ga_5_O_12_/Au-NP sample is ~100 nm, which is comparable to that in the Pt/BiY_2_Fe_5_O_12_/Au-NP sample.

### Numerical calculation

To investigate the electromagnetic field distributions induced by surface plasmons, we performed the numerical calculation based on the FDTD method^25^ using KeyFDTD (Science Technology Research Institute, Japan). The simulation results shown in Fig. 3 were obtained from a model comprising a BiY_2_Fe_5_O_12_ rectangular parallelepiped with the size of 150 nm × 150 nm × 140 nm and a Au spheroid with the in-plane diameter *d*_Au_ of 70 nm and the height *h*_Au_ of 40 nm embedded at the centre of the BiY_2_Fe_5_O_12_, where the distance *L* between the tops of the BiY_2_Fe_5_O_12_ and the Au is 50 nm. The unit cell of the model is 2 nm × 2 nm × 1 nm. A flat light source is placed on the top of the BiY_2_Fe_5_O_12_ rectangular parallelepiped in the *x*-*y* plane, which generates plane electromagnetic waves with the polarization along the *x* direction and the wave vector **k** along the *z* direction (note that the incident light used in the experiments is unpolarized). Here, the *x*, *y* and *z* directions are defined in Fig. 3a. At the outer boundaries of the BiY_2_Fe_5_O_12_, we set a periodic boundary condition. Under this condition, we calculated the distribution of the electric field intensity |*E*| while fixing the wavelength of the incident electromagnetic waves. In Fig. 3b,c, we plot the |*E*| distribution in the BiY_2_Fe_5_O_12_/Au-NP model in the *x*-*y* plane across the centre of the Au spheroid. By using the FDTD method and similar models, we have also investigated the electromagnetic coupling between the Pt film and Au NPs in the Pt/BiY_2_Fe_5_O_12_/Au-NP sample, which might affect the voltage signal under the SPR condition, by comparing the simulated distributions of |*E*| induced by the SPR between the BiY_2_Fe_5_O_12_/Au-NP models with and without the Pt layer for various values of *L*, *d*_Au_ and *h*_Au_ (Supplementary Figs 1 and 2). In Supplementary Fig. 5, we compare the simulated |*E*| distributions between the Pt/BiY_2_Fe_5_O_12_/Au-NP models with and without a void. The refraction index and absorption-constant spectra of BiY_2_Fe_5_O_12_, Au and Pt in the calculations were obtained from refs 37, 38, 39, respectively.

## Author contributions

K.U. and E.S. planned and supervised the study. K.U. designed the experiments, prepared the samples, collected and analysed all of the data and performed the numerical calculation. H.A. and S.M. developed the explanation of the experiments. D.K. performed the scanning electron microscopy. S.I. performed the transmission electron microscopy. Z.Q. supported the sample preparation. K.U. and H.A. wrote the manuscript with input from E.S. and S.M. All authors discussed the results.

## Additional information

**How to cite this article:** Uchida, K. *et al*. Generation of spin currents by surface plasmon resonance. *Nat. Commun.* 6:5910 doi: 10.1038/ncomms6910 (2015).

## Supplementary Material

Supplementary Information Supplementary Figures 1-6, Supplementary Notes 1-2, and Supplementary References
