# Supplementary material for: Generation of spin currents by surface plasmon resonance
Source: Nat Commun. 2015 Jan 8;6:5910. doi: 10.1038/ncomms6910 (PMC4354158; doi:10.1038/ncomms6910)
Supplement: Supplementary Information — Supplementary Figures 1-6, Supplementary Notes 1-2, and Supplementary References [file ncomms6910-s1.pdf]

Supplementary Figures

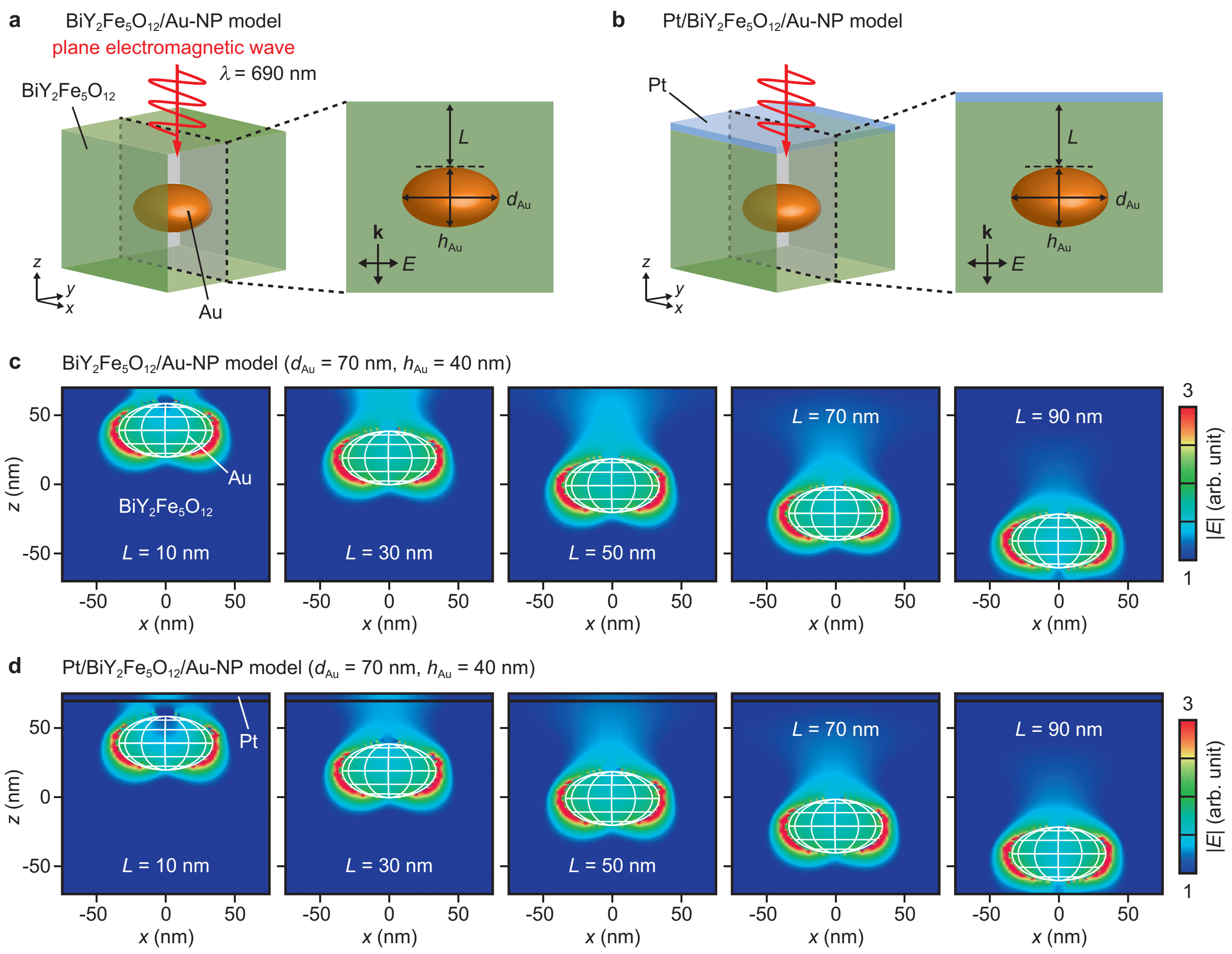

Supplementary Figure 1 | Dependence of electric field distribution on the position of Au nanoparticles. **a**,**b**, Schematic illustrations of the $BiY_2Fe_5O_{12}$/Au-NP (**a**) and Pt/$BiY_2Fe_5O_{12}$/Au-NP (**b**) models used for the finite-difference time-domain (FDTD) simulation. The $BiY_2Fe_5O_{12}$/Au-NP model comprises a $BiY_2Fe_5O_{12}$ rectangular parallelepiped with the size of 150 nm × 150 nm × 140 nm and a Au spheroid with the in-plane diameter d_{Au} and the height h_{Au} embedded at the centre of the $BiY_2Fe_5O_{12}$, where L is the distance between the tops of the $BiY_2Fe_5O_{12}$ and the Au. The Pt/$BiY_2Fe_5O_{12}$/Au-NP model consists of the $BiY_2Fe_5O_{12}$/Au-NP model and a Pt rectangular parallelepiped with the size of 150 nm × 150 nm × 5 nm attached on the top of the $BiY_2Fe_5O_{12}$. **c**,**d**, Simulated distributions of the electric field intensity $|E|$ in the $BiY_2Fe_5O_{12}$/Au-NP (**c**) and Pt/$BiY_2Fe_5O_{12}$/Au-NP (**d**) models at $\lambda = 690$ nm in the z-x plane across the centre of the Au spheroid for various values of L, calculated when $d_{Au} = 70$ nm and $h_{Au} = 40$ nm. In both the models, strong $|E|$ is induced in the vicinity of the $BiY_2Fe_5O_{12}$/Au-NP interface due to the localized surface plasmon resonance (SPR). Importantly, the $|E|$ distributions in $BiY_2Fe_5O_{12}$ are not affected by the presence of Pt irrespective of the distance between the Pt and Au NP. Note that the values of L used in the calculations are smaller than the mean distance between the Pt film and Au NPs in the experiments.

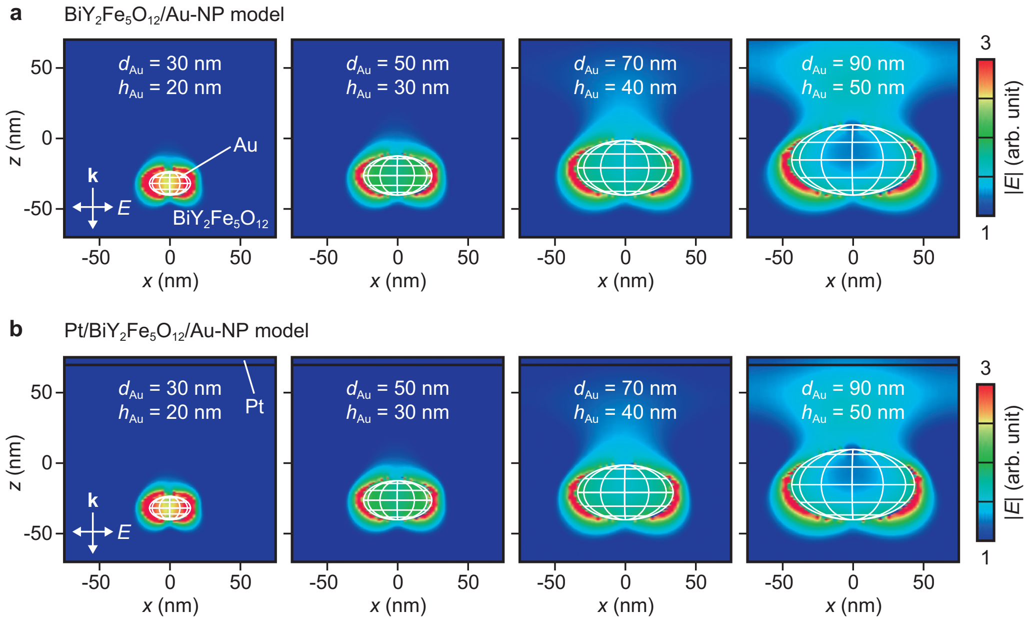

Supplementary Figure 2 | Dependence of electric field distribution on the size of Au nanoparticles. **a**,**b**, Simulated $|E|$ distributions in the $BiY_2Fe_5O_{12}$/Au-NP (**a**) and Pt/$BiY_2Fe_5O_{12}$/Au-NP (**b**) models at λ = 690 nm in the z-x plane across the centre of the Au spheroid for various values of d_{Au} and h_{Au}. These simulation results confirm that, although the $|E|$ distributions in $BiY_2Fe_5O_{12}$ under the SPR condition depend on the size of the Au NP, they are not affected by the presence of Pt irrespective of the values of d_{Au} and h_{Au}.

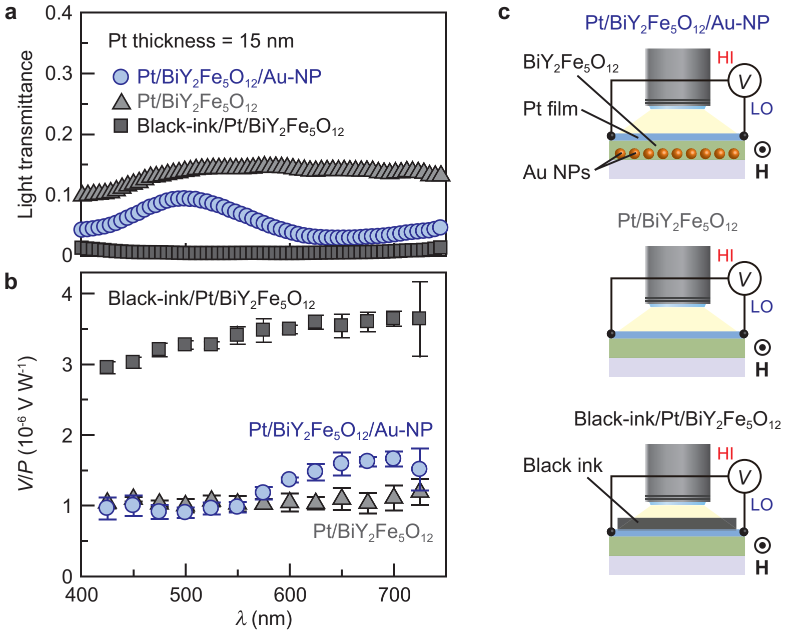

Supplementary Figure 3 | Measurements using thicker Pt films. **a**, λ dependence of the light transmittance of the Pt(15 nm)/$BiY_2Fe_5O_{12}$/Au-NP, Pt(15 nm)/$BiY_2Fe_5O_{12}$, and Black-ink/Pt(15 nm)/$BiY_2Fe_5O_{12}$ samples in which the 5-nm-thick Pt films are replaced with 15-nm-thick Pt films. The $BiY_2Fe_5O_{12}$ layers of the samples were prepared by the same process as the contacted Pt/$BiY_2Fe_5O_{12}$/Au-NP sample. Due to the thicker Pt films, the light transmittance of these samples is much smaller than that of the samples used in the main text. When the surface of the Pt(15 nm)/$BiY_2Fe_5O_{12}$ sample is coated with black ink, the transmission of light and the resultant interaction between photons and magnons in $BiY_2Fe_5O_{12}$ are almost completely blocked. **b**, λ dependence of V/P, measured when the external magnetic field $\mathbf{H}$ of the magnitude $H = 200$ Oe was applied along the x direction. We observed finite V/P signals at all the λ values in the Pt(15 nm)/$BiY_2Fe_5O_{12}$ sample without Au NPs (see gray triangle data points) and found that its λ dependence is similar to the result for the Pt(5 nm)/$BiY_2Fe_5O_{12}$ sample without Au NP. Similar signals appear also in the Black-ink/Pt(15 nm)/$BiY_2Fe_5O_{12}$ sample. These results confirm that the background spin-current signals in the Pt/$BiY_2Fe_5O_{12}$ samples without Au NPs are attributed to the heating of the samples, or the longitudinal spin Seebeck effect (LSSE) (note that the enhancement of the heating signal in the Black-ink/Pt(15 nm)/$BiY_2Fe_5O_{12}$ sample is due to the larger temperature rise caused by light absorption by the black ink). In the Pt(15 nm)/$BiY_2Fe_5O_{12}$/Au-NP sample, we observed the voltage enhancement due to the plasmonic spin pumping under the SPR condition (see blue circle data points). The voltage enhancement observed here is weaker than that in the Pt(5 nm)/$BiY_2Fe_5O_{12}$/Au-NP sample because the thicker Pt layer blocks the transmission of light and reduces the magnitude of the near fields in the $BiY_2Fe_5O_{12}$ layer, a situation which changes the relative magnitude between the plasmonic-spin-pumping signal and the background LSSE signal. **c**, Experimental configuration and sample structure for measuring voltage in the Pt(15 nm)/$BiY_2Fe_5O_{12}$/Au-NP, Pt(15 nm)/$BiY_2Fe_5O_{12}$, and Black-ink/Pt(15 nm)/$BiY_2Fe_5O_{12}$ samples.

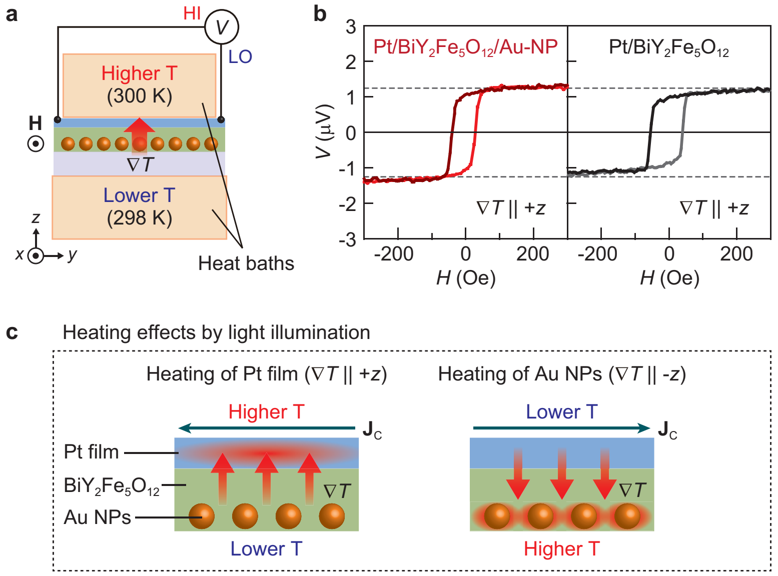

Supplementary Figure 4 | Sign of voltage signals under temperature gradients. **a**, Experimental configuration for measuring the LSSE in the Pt/$BiY_2Fe_5O_{12}$/Au-NP and Pt/$BiY_2Fe_5O_{12}$ samples. The LSSE was measured by sandwiching the sample between two heat baths of which the temperatures are stabilized at 300 K and 298 K, where the heat bath of 300 K (298 K) is thermally connected to the top of the Pt film (the bottom of the substrate); the temperature gradient ∇T is along the $+z$ direction. Here, **H** was applied to the samples along the x direction. Note that the samples are not illuminated during the LSSE measurements. **b**, H dependence of V in the Pt/$BiY_2Fe_5O_{12}$/Au-NP and Pt/$BiY_2Fe_5O_{12}$ samples, measured when the Pt layer is hotter than the $BiY_2Fe_5O_{12}$ layer ($\nabla T \parallel +z$). The dark red and black lines (light red and gray lines) show the V signals measured when **H** was swept from positive to negative (from negative to positive). **c**, The directions of the temperature gradient induced by the light illumination in the Pt/$BiY_2Fe_5O_{12}$/Au-NP sample. The ∇T direction and the sign of the LSSE voltage due to the heating of Au NPs under the SPR condition are opposite to those due to the heating of the Pt film.

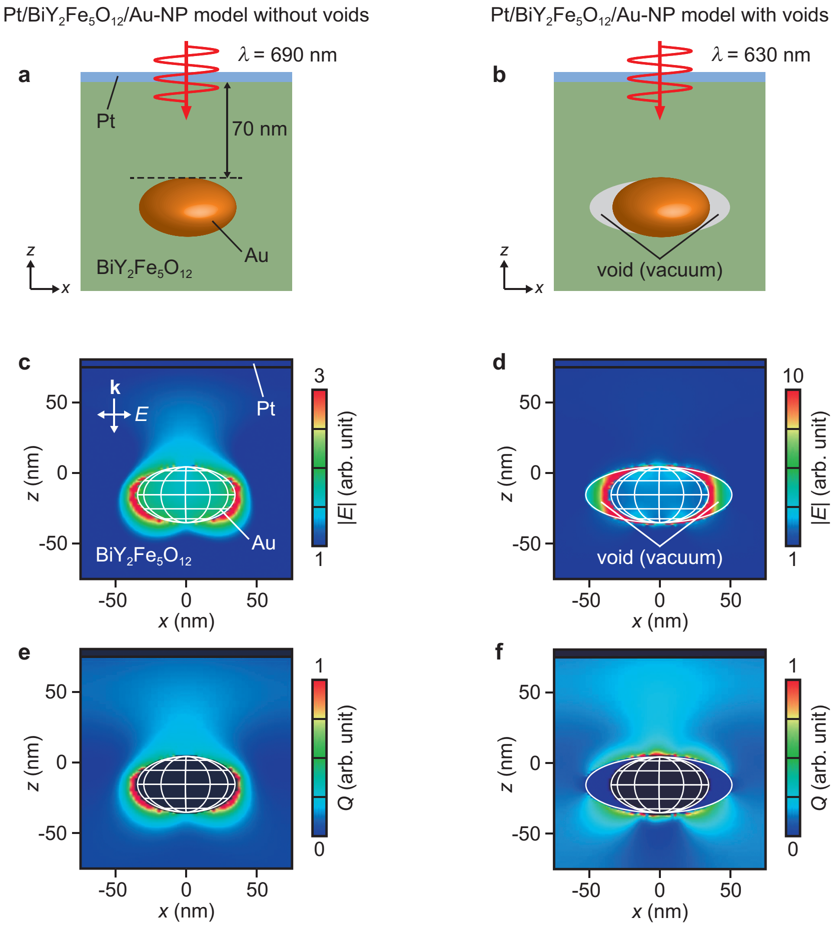

Supplementary Figure 5 | Effect of voids. **a**,**b**, Schematic illustrations of the Pt/$BiY_2Fe_5O_{12}$/Au-NP models without and with a void used for the FDTD simulation. To model the void, a vacuum spheroid with the in-plane diameter of 100 nm and the height of 40 nm is added to the Pt/$BiY_2Fe_5O_{12}$/Au-NP model around the Au spheroid, where $L = 70$ nm, $d_{\mathrm{Au}} = 70$ nm, and $h_{\mathrm{Au}} = 40$ nm. According to the experimental results in the main text, the wavelength of the incident electromagnetic waves is fixed at $\lambda = 690$ nm ($\lambda = 630$ nm) for the Pt/$BiY_2Fe_5O_{12}$/Au-NP model without (with) the void. **c**,**d**, Simulated distributions of $|E|$ in the Pt/$BiY_2Fe_5O_{12}$/Au-NP models without and with the void in the z-x plane across the centre of the Au spheroid. In the model without the void, the strong near fields are induced in $BiY_2Fe_5O_{12}$ in the vicinity of the Au spheroid due to the SPR. In contrast, in the model with the void, most of the photon energy is confined in the void, indicating that the plasmon-induced near fields cannot interact with magnons in $BiY_2Fe_5O_{12}$ with voids. These simulation results are consistent with our experiments, where the plasmonic spin pumping appears in the contacted Pt/$BiY_2Fe_5O_{12}$/Au-NP sample while only heating effect appears in the voided sample. **e**,**f**, Simulated distributions of the power dissipation Q in the Pt/$BiY_2Fe_5O_{12}$/Au-NP models without and with the void in the z-x plane across the centre of the Au spheroid. The maximum of Q is in the vicinity of the Au spheroid in both the models, indicating that the heating due to the SPR is induced near Au NPs irrespective of the presence of voids, allowing us to conclude that the temperature gradient induced by the SPR is of an opposite sign to that induced by the heating of the Pt layer in both the contacted and voided Pt/$BiY_2Fe_5O_{12}$/Au-NP samples.

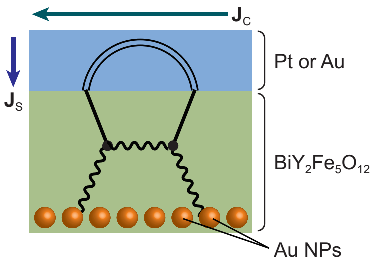

Supplementary Figure 6 | Feynman diagram for calculating the spin current generated by the plasmonic spin pumping. The wavy lines represent a (near-field) photon propagator or external photon field. The double line and bold lines represent itinerant-spin-density and magnon propagators, respectively.

Supplementary Note 1

To further discuss the heating effects, we measured the LSSE in the contacted Pt/$BiY_2Fe_5O_{12}$/Au-NP and Pt/$BiY_2Fe_5O_{12}$ samples. As shown in Supplementary Fig. 4a,b, both the samples exhibit clear LSSE voltage; when the temperature gradient is generated along the $+z$ direction, the positive (negative) V signal appears at positive (negative) H values in the present configuration. These LSSE data confirm that the temperature of the Pt layer is higher than that of the $BiY_2Fe_5O_{12}$ layer under the light illumination in the configuration shown in Fig. 1a in the main text, since the sign of the background signals in Figs 4 and 8 in the main text is the same as that of the LSSE voltage observed here. Importantly, as shown in Supplementary Fig. 4b, the magnitude of the LSSE voltage in the Pt/$BiY_2Fe_5O_{12}$/Au-NP sample is comparable to that in the Pt/$BiY_2Fe_5O_{12}$ sample. In the experiments shown in the main text, since both the Pt/$BiY_2Fe_5O_{12}$/Au-NP and Pt/$BiY_2Fe_5O_{12}$ samples are illuminated from the Pt-film side, the temperature rise of the Pt layer and the resultant background LSSE voltage are almost the same for both. Therefore, we can conclude that the heating of the Pt layer is irrelevant to the voltage enhancement under the SPR condition in the contacted Pt/$BiY_2Fe_5O_{12}$/Au-NP sample.

The sign of the voltage signal induced by the LSSE voltage is determined by the direction of the temperature gradient across the Pt/$BiY_2Fe_5O_{12}$ interface, not by the total amount of heat generation. Here, we emphasize again that the direction of the temperature gradient and the sign of the LSSE voltage due to the heating of Au NPs under the SPR condition are opposite to those due to the heating of the Pt film (Supplementary Fig. 4c). Although our samples have substantial variations in the size, position, and shape of Au NPs, the Au NPs are in contact with the substrate and coated with the $BiY_2Fe_5O_{12}$ film (see Fig. 7 in the main text). Therefore, the situation illustrated in Supplementary Fig. 4c can never be changed by the variations of Au NPs. Nevertheless, the magnitude of the V signal in the contacted Pt/$BiY_2Fe_5O_{12}$/Au-NP sample is strongly enhanced compared to that in the Pt/$BiY_2Fe_5O_{12}$ sample under the SPR condition (see Fig. 4b in the main text). This result cannot be explained by the LSSE voltage induced by the heating of Au NPs because of the opposite sign, requiring us to introduce nonequilibrium magnons excited by surface-plasmon-induced evanescent electromagnetic fields in the $BiY_2Fe_5O_{12}$ layer (see Supplementary Note 2)[*1][*2].

[*1] The LSSE voltage induced by a heat current flowing from a magnetic insulator to a metallic contact is opposite in sign to the inverse spin Hall voltage induced by magnon excitation in the magnetic insulator, since the heat current strongly excites conduction-electron spins in the metallic contact, as demonstrated by the studies on the LSSE and the acoustic spin pumping[1-4].

[*2] The small enhancement of the voltage around 400 nm in the contacted Pt/$BiY_2Fe_5O_{12}$/Au-NP sample in Fig. 4b in the main text also cannot be explained by the heating of the $BiY_2Fe_5O_{12}$ layer because of the different sign. Although this enhancement might be related to the absorption of photons in the $BiY_2Fe_5O_{12}$ layer in the short-wavelength range, its origin remains to be clarified.

Supplementary Note 2

Here, we formulate a linear response theory of the plasmonic spin pumping and show that the experimental observation can be explained as a consequence of the energy transfer from plasmon-induced near-field photons to magnons in the $BiY_2Fe_5O_{12}$ layer. Interaction of photons and magnons has been extensively studied in the context of Raman scattering by magnons[5-8]. From these studies, it has been established that the dominant coupling mechanism is given by an electric-dipole coupling via a spin-orbit interaction. Because the energy of incident near-field photons (1.6-3.1 eV) is much higher than the energy scale of magnons (< 0.1 eV), a linear coupling between photons and magnons via a direct magnetic-dipole interaction[9], i.e., optical generation of magnons by light absorption, is irrelevant to the present discussion, as discussed in ref. 8.

Following ref. 10, the Hamiltonian that gives rise to the scattering of light by a magnetic system is written as

$$H' = \sum_{j_1,j_2} \sum_{\boldsymbol{r}_i} E_1^{j_1}(\boldsymbol{r}_i) E_2^{j_2}(\boldsymbol{r}_i) \Pi^{j_1,j_2}(\boldsymbol{r}_i), \tag{1}$$

where $\boldsymbol{E}_{1(2)}$ is the incident (scattered) electric field at position $\boldsymbol{r}_i$, $j_{1(2)}$ represents a Cartesian component of the field vector, and $\Pi^{j_{1(2)}}(\boldsymbol{r}_i)$, a term containing the spin operator $\boldsymbol{S}(\boldsymbol{r}_i)$, describes the spin-dependent polarizability tensor at $\boldsymbol{r}_i$. For systems such as $BiY_2Fe_5O_{12}$, equation (1) can be expressed in the form[11]

$$H' = \frac{v_0}{2} \sum_{\boldsymbol{r}_i} \Big(\big[\Gamma^- E_1^z(\boldsymbol{r}_i) E_2^-(\boldsymbol{r}_i) + \Gamma^+ E_1^-(\boldsymbol{r}_i) E_2^z(\boldsymbol{r}_i)\big] S^-(\boldsymbol{r}_i) + \big[\Gamma^+ E_1^z(\boldsymbol{r}_i) E_2^+(\boldsymbol{r}_i) + \Gamma^- E_1^-(\boldsymbol{r}_i) E_2^z(\boldsymbol{r}_i)\big] S^+(\boldsymbol{r}_i) \Big) \tag{2}$$

to the lowest order in the spin operators, where $S^\pm = S^x \pm \mathrm{i}S^y$, $E^\pm = E^x \pm \mathrm{i}E^y$, and $v_0 = \mathcal{V}/N_0$ is the effective block spin volume with the number of unit cells N_0. In the above equation, $\Gamma^\pm = G \pm \mathrm{i}M$ is the dimensionless magnon-photon coupling constant, where we assume that G and M are real numbers. Note that the magnon-photon coupling in equation (2) forms the basis for the Brillouin light scattering investigation of $Y_3Fe_5O_{12}$ (ref. 12). The incident electric field $\boldsymbol{E}_1$ is an external c-number field with the representation

$$\boldsymbol{E}_1(\boldsymbol{r}, t) = \sum_{\zeta_1} \sum_{\boldsymbol{K}_1 = \pm \boldsymbol{K}_0} \boldsymbol{E}_{\boldsymbol{K}_1,\zeta_1}(t) e^{\mathrm{i}\boldsymbol{K}_1 \cdot \boldsymbol{r}}, \tag{3}$$

where the coefficient satisfies $\boldsymbol{E}_{-\boldsymbol{K}_1,\zeta_1}(t) = \boldsymbol{E}^*_{\boldsymbol{K}_1,\zeta_1}(t)$ and is expressed as $\boldsymbol{E}_{\boldsymbol{K}_1,\zeta_1}(t) = \boldsymbol{\xi}(\boldsymbol{K}_1, \zeta_1)\mathcal{E}_{\boldsymbol{K}_1,\zeta_1}$ with the wavenumber $\boldsymbol{K}_1$, polarization ζ_1, and amplitude $\mathcal{E}_{\boldsymbol{K}_1,\zeta_1}$. The scattered electric field $\boldsymbol{E}_2$ is, on the other hand, expressed in a quantized form[10]. It is custom to express this field by using the vector potential $\boldsymbol{A}_2$ as $\boldsymbol{E}_2(\boldsymbol{r}, t) = -c^{-1}\partial_t \boldsymbol{A}_2(\boldsymbol{r}, t)$, where c is the velocity of light. The vector potential is represented as

$$\boldsymbol{A}_2(\boldsymbol{r}, t) = \sum_{\zeta_2} \sum_{\boldsymbol{K}_2} A_{\boldsymbol{K}_2,\zeta_2}(t) e^{\mathrm{i}\boldsymbol{K}_2 \cdot \boldsymbol{r}}, \tag{4}$$

where $\boldsymbol{A}_{\boldsymbol{K}_2,\zeta_2}(t) = \sqrt{\frac{2\pi\hbar}{\nu_{\boldsymbol{K}_2}\mathcal{V}}} \boldsymbol{\xi}(\boldsymbol{K}_2, \zeta_2)[a_{\boldsymbol{K}_2,\zeta_2}(t) + a^\dagger_{-\boldsymbol{K}_2,\zeta_2}(t)]$ with the photon frequency $\nu_{\boldsymbol{K}} = cK$, the photon annihilation and creation operators $a_{\boldsymbol{K}_2,\zeta_2}$ and $a^\dagger_{-\boldsymbol{K}_2,\zeta_2}$, and the polarization vector $\boldsymbol{\xi}(\boldsymbol{K}_2, \zeta_2)$. Using the linear spin-wave approximation

$$S^+(\boldsymbol{r}_i) = \sqrt{\frac{2S_0}{N_0}} \sum_{\boldsymbol{q}} b_{\boldsymbol{q}} e^{\mathrm{i}\boldsymbol{q} \cdot \boldsymbol{r}} \tag{5}$$

and

$$S^{-}(\boldsymbol{r}_i) = \sqrt{\frac{2S_0}{N_0}} \sum_{\boldsymbol{q}} b_{\boldsymbol{q}}^{\dagger} e^{-\mathrm{i}\boldsymbol{q}\cdot\boldsymbol{r}} \tag{6}$$

with the size of the localized spin S_0, the magnon-photon interaction is represented as

$$H' = -\frac{1}{c}\sqrt{\frac{S_0 v_0}{2}} \sum_{\boldsymbol{q}} \sum_{\boldsymbol{K}_1=\pm\boldsymbol{K}_0} \Big(\Gamma^{-} E^{z}_{-\boldsymbol{K}_1}\big(\partial_t A^{-}_{\boldsymbol{K}_1-\boldsymbol{q}}\big) + \Gamma^{+} E^{-}_{-\boldsymbol{K}_1}\big(\partial_t A^{z}_{\boldsymbol{K}_1-\boldsymbol{q}}\big)\Big) b_{\boldsymbol{q}} + H.c., \tag{7}$$

where $H.c.$ means the Hermitian conjugate. In the present situation, the incident electric field $\boldsymbol{E}_1$ comes from the near-field photons induced by the localized SPR in the Au NPs embedded in the $BiY_2Fe_5O_{12}$ film. Therefore, we assume that its polarization direction as well as its propagating direction should be averaged out in the final step of our calculation.

Following the formalism developed in ref. 3, we now calculate the spin current J_{S} generated by the plasmonic spin pumping. We consider a model shown in Supplementary Fig. 6, and investigate the spin injection from a ferrimagnetic insulator (FI, in the experiments $BiY_2Fe_5O_{12}$) into an attached paramagnetic metal (PM, in the experiments Pt or Au) through the *s*-*d* exchange interaction acting at the PM/FI interface:

$$\mathcal{H}_{\mathrm{sd}} = J_{\mathrm{sd}} \sum_{\boldsymbol{r}_0\in\mathrm{interface}} \boldsymbol{s}(\boldsymbol{r}_0)\cdot\boldsymbol{S}(\boldsymbol{r}_0), \tag{8}$$

where $\boldsymbol{s}$ is the conduction-electrons' spin density in PM, $\boldsymbol{S}$ is the localized spin in FI, and J_{sd} is the strength of the interface *s*-*d* exchange coupling. The spin current J_{S} generated in PM can be calculated as a rate of change of the spin density in PM as $J_{\mathrm{S}} = \frac{\hbar}{2}\sum_{\boldsymbol{r}_i\in\mathrm{PM}}\langle\partial_t \boldsymbol{s}(\boldsymbol{r}_i,t)\rangle$, where $\langle\cdots\rangle$ denotes the statistical average at a given time t. Assuming that the spin-orbit interaction is weak enough in the neighborhoods of the interface, the Heisenberg equation of motion for $\boldsymbol{s}$ yields

$$J_{\mathrm{S}} = \sum_{\boldsymbol{q},\boldsymbol{k}} \frac{-2\mathcal{J}_{\mathrm{sd}}^{\boldsymbol{k}-\boldsymbol{q}}\sqrt{S_0}}{\sqrt{2N_{\mathrm{P}}N_{\mathrm{F}}}} \int_{-\infty}^{\infty} \frac{d\omega}{2\pi} \mathrm{Re}C^{<}_{\boldsymbol{k},\boldsymbol{q}}(\omega), \tag{9}$$

where $\mathcal{J}_{\mathrm{sd}}^{\boldsymbol{k}-\boldsymbol{q}}$ is the Fourier transform of $\mathcal{J}_{\mathrm{sd}}(\boldsymbol{r}) = J_{\mathrm{sd}}\sum_{\boldsymbol{r}_0\in\mathrm{interface}} a_{\mathrm{S}}^3\delta(\boldsymbol{r}-\boldsymbol{r}_0)$, $N_{\mathrm{P(F)}}$ is the number of lattice sites in PM (FI), and we represent the effective block spin volume as $v_0 = a_{\mathrm{S}}^3$ by introducing a_{S}. Here, $C^{<}_{\boldsymbol{k},\boldsymbol{q}}(\omega)$ is the Fourier transform of the interface correlation $C^{<}_{\boldsymbol{k},\boldsymbol{q}}(t,t') = -\mathrm{i}\langle b^{+}_{\boldsymbol{q}}(t')s^{-}_{\boldsymbol{k}}(t)\rangle$ between the magnons and the spin density $s^{-}_{\boldsymbol{k}} = \frac{1}{2\sqrt{N_{\mathrm{P}}}}\sum_{\boldsymbol{r}_i}[s^x(\boldsymbol{r}_i) - \mathrm{i}s^y(\boldsymbol{r}_i)]e^{-\mathrm{i}\boldsymbol{k}\cdot\boldsymbol{r}_i}$.

The process relevant to the plasmonic spin pumping is shown in Supplementary Fig. 6; in the case of the plasmon-induced spin injection, the near-field photons concomitant with surface plasmons excite only magnons in the $BiY_2Fe_5O_{12}$ film since they are localized in the vicinity of the $BiY_2Fe_5O_{12}$/Au-NP interface (see Fig. 3 in the main text and Supplementary Figs 1, 2, and 5). Due to the similarity between this diagram and that for the acoustic spin pumping (ASP)[1,2] (Fig. 10 of ref. 3), the present calculation for the plasmonic spin pumping is mostly the same as that for the ASP, with the replacement of external *phonon* lines by external *photon* lines. One big difference is that the intermediate state is given by scattered photons in the present situation whereas it is given by magnons for the ASP. This is because the energy of external photons (1.6-3.1 eV) is much larger than that of magnons ($<$ 0.1 eV), and most of the external photon energy contributing to this process is transferred to scattered photons, leaving a small energy transfer to magnons.

Using the same procedure as in ref. 3, the spin current generated by the process shown in Supplementary Fig. 6 is calculated to be

$$J_{\mathrm{S}} = \frac{\sqrt{2} N_{\mathrm{int}} J_{\mathrm{sd}}^2 S_0^2}{2\hbar^3 N_{\mathrm{P}} N_{\mathrm{F}}} \left(v_0 |\mathcal{E}_{\boldsymbol{K}_0}|^2 \right) \sum_{\boldsymbol{k},\boldsymbol{q}} \Gamma^+ \Gamma^- B_{\boldsymbol{k},\boldsymbol{q}}(\nu_{\boldsymbol{K}_0}), \tag{10}$$

where N_{int} is the number of localized spins in FI at the interface. The quantity $B_{\boldsymbol{k},\boldsymbol{q}}(\nu_{\boldsymbol{K}_0})$ is defined by

$$\begin{aligned} B_{\boldsymbol{k},\boldsymbol{q}}(\nu_{\boldsymbol{K}_0}) = \left(\frac{\nu_{\boldsymbol{K}_0}}{c} \right)^2 \int_\omega \mathrm{Im}\chi_{\boldsymbol{k}}^{\mathrm{R}}(\omega) \mathrm{Im} D_{\boldsymbol{q}-\boldsymbol{K}_0}^{\mathrm{R}}(\omega - \nu_{\boldsymbol{K}_0}) |X_{\boldsymbol{q}}^{\mathrm{R}}(\omega)|^2 \left[\coth\left(\frac{\hbar(\omega - \nu_{\boldsymbol{K}_0})}{2k_{\mathrm{B}}T} \right) - \coth\left(\frac{\hbar\omega}{2k_{\mathrm{B}}T} \right) \right] \\ + (\nu_{\boldsymbol{K}_0} \to -\nu_{\boldsymbol{K}_0};\, \boldsymbol{K}_0 \to -\boldsymbol{K}_0) \end{aligned} \tag{11}$$

with the shorthand notation $\int_\omega = \int_{-\infty}^{\infty} \frac{d\omega}{2\pi}$. In the above equation, $\chi_{\boldsymbol{k}}^{\mathrm{R}}(\omega) = \chi_{\mathrm{P}}/(1 + \lambda_{\mathrm{sf}}^2 k^2 - \mathrm{i}\omega\tau_{\mathrm{sf}})$ is the retarded component of the itinerant-spin-density propagator in PM with χ_{P}, λ_{sf}, and τ_{sf} being respectively the paramagnetic susceptibility, spin diffusion length, and spin relaxation time. Also, $X_{\boldsymbol{q}}^{\mathrm{R}}(\omega) = (\omega - \widetilde{\omega}_{\boldsymbol{q}} + \mathrm{i}\alpha\omega)^{-1}$ is the retarded component of the magnon propagator with $\widetilde{\omega}_{\boldsymbol{q}} = \gamma H_0 + \omega_{\boldsymbol{q}}$ and α being respectively the magnon frequency and Gilbert damping constant, and $D_{\boldsymbol{K}}^{\mathrm{R}}(\nu) = (4\pi\hbar c^2/2\nu_{\boldsymbol{K}})[(\nu - \nu_{\boldsymbol{K}} + \mathrm{i}0^+)^{-1} - (\nu + \nu_{\boldsymbol{K}} + \mathrm{i}0^+)^{-1}]$ is the retarded component of the photon propagator. Integrating over ω by picking up the magnon poles and using the fact that the dominant contribution comes from a region $q \ll K_0$, $B_{\boldsymbol{k},\boldsymbol{q}}(\nu_{\boldsymbol{K}_0})$ is calculated to be

$$B_{\boldsymbol{k},\boldsymbol{q}}(\nu_{\boldsymbol{K}_0}) \approx -\frac{2\pi^2\hbar\nu_{\boldsymbol{K}_0}}{cq\alpha} \delta\left(\hat{\boldsymbol{K}}_0 \cdot \hat{\boldsymbol{q}} - \frac{\omega_q}{\nu_q} \right) \left(\frac{1}{\omega_{\boldsymbol{q}}} \mathrm{Im}\chi_{\boldsymbol{k}}^{\mathrm{R}}(\omega_{\boldsymbol{q}}) \right), \tag{12}$$

where $\hat{\boldsymbol{K}}_0 = \boldsymbol{K}_0/K_0$ and $\hat{\boldsymbol{q}} = \boldsymbol{q}/q$. Therefore, the spin current pumped by light illumination is finally given by

$$J_{\mathrm{S}} = \Gamma^+\Gamma^- G_{\mathrm{S}} \frac{\hbar\nu_{\boldsymbol{K}_0} a_{\mathrm{S}}^4}{c\alpha} |\mathcal{E}_{\boldsymbol{K}_0}|^2, \tag{13}$$

where $G_{\mathrm{S}} = -\frac{\sqrt{2} N_{\mathrm{int}} J_{\mathrm{sd}}^2 S_0^2 \chi_{\mathrm{P}} \tau_{\mathrm{sf}}}{16\pi^2\hbar^3(\lambda_{\mathrm{sf}}/a)^3} \Upsilon$ is a factor measuring the strength of the magnetic coupling at the PM/FI interface (corresponding to the spin mixing conductance), a is the lattice constant of PM, and $\Upsilon = \int_0^1 dx \int_0^{a_{\mathrm{S}}K_0} dy \frac{x^2}{(1+x^2)^2 + (2S_0 J_{\mathrm{ex}} \tau_{\mathrm{sf}}/\hbar)^2 y^2} \approx 0.142 a_{\mathrm{S}} K_0$ with the strength of the exchange coupling J_{ex} in FI and the dimensionless variables $x = \lambda_{\mathrm{sf}} k$ and $y = \hbar\omega_{\boldsymbol{q}}/(2J_{\mathrm{ex}}S_0)$. In equation (2) in the main text, for simplicity, we describe $\nu_{\boldsymbol{K}_0}$ and $\mathcal{E}_{\boldsymbol{K}_0}$ as ν and E, respectively. Equation (13) represents a physical process in which magnons in FI is excited by a small energy transfer from external photons, thereby having the same sign as that for the ASP[1,2,*3]. This situation is consistent with the experimental results of the ASP in a Pt/$Y_3Fe_5O_{12}$ structure (e.g., Fig. 4d of ref. 1), where the sign of the background LSSE signal owing to the heating of $Y_3Fe_5O_{12}$ is opposite to that of the ASP signal owing to the vibration of $Y_3Fe_5O_{12}$. Since the direction of the temperature gradient across the Pt/$BiY_2Fe_5O_{12}$ interface in the present experimental configuration is opposite to that in the ASP configuration, the voltage signal coming from the plasmonic spin pumping has the same sign as the background LSSE signal (see Fig. 4b in the main text). Note that, theoretically, there can be another plasmonic spin pumping process in which the plasmon-enhanced near fields first excite phonons through phonon Raman scattering and then the resultant phonons drive the spin

[*3] Note that the definition of the voltage V in this paper is opposite in sign to that in our previous literature on the ASP.

pumping via magnon-phonon interaction. This higher-order process can be taken into account with the replacement $\Gamma^+\Gamma^- \to \Lambda^2\Upsilon^2$ and $B_{\boldsymbol{k},\boldsymbol{q}}(\nu_{\boldsymbol{K}_0}) \to B'_{\boldsymbol{k},\boldsymbol{q}}(\nu_{\boldsymbol{K}_0})$, where Λ is the phonon-photon Raman scattering vertex, Υ is the magnon-phonon coupling constant, and

$$B'_{\boldsymbol{k},\boldsymbol{q}}(\nu_{\boldsymbol{K}_0}) = \left(\frac{\nu_{\boldsymbol{K}_0}}{c}\right)^2 \sum_{\boldsymbol{p}} \int_\omega \int_\varepsilon \mathrm{Im}\chi^{\mathrm{R}}_{\boldsymbol{k}}(\omega)\mathrm{Im}X^{\mathrm{R}}_{\boldsymbol{q}-\boldsymbol{p}}(\omega-\varepsilon)|X^{\mathrm{R}}_{\boldsymbol{q}}(\omega)|^2|\mathcal{D}^{\mathrm{R}}_{\boldsymbol{p}}(\varepsilon)|^2\mathrm{Im}D^{\mathrm{R}}_{\boldsymbol{p}-\boldsymbol{K}_0}(\varepsilon-\nu_{\boldsymbol{K}_0})$$
$$\times\left[\coth\left(\frac{\hbar(\omega-\varepsilon)}{2k_{\mathrm{B}}T}\right)-\coth\left(\frac{\hbar\omega}{2k_{\mathrm{B}}T}\right)\right]\left[\coth\left(\frac{\hbar(\varepsilon-\nu_{\boldsymbol{K}_0})}{2k_{\mathrm{B}}T}\right)-\coth\left(\frac{\hbar\varepsilon}{2k_{\mathrm{B}}T}\right)\right]$$
$$+(\nu_{\boldsymbol{K}_0}\to-\nu_{\boldsymbol{K}_0};\ \boldsymbol{K}_0\to-\boldsymbol{K}_0) \qquad (14)$$

with $\mathcal{D}^{\mathrm{R}}_{\boldsymbol{p}}(\varepsilon) = [(\varepsilon-\varepsilon_{\boldsymbol{p}}+\mathrm{i}0^+)^{-1} - (\varepsilon+\varepsilon_{\boldsymbol{p}}+\mathrm{i}0^+)^{-1}]$ being the retarded component of a phonon propagator. Both these two processes can in principle contribute to the plasmonic spin pumping.

As an endnote to Supplementary Note 2, we mention the effect of the anisotropy of magneto-optical coefficients in $BiY_2Fe_5O_{12}$. According to Table 2 in Ref. 11, the magnitude of the anisotropy in magnetic linear birefringence is estimated to be at most 50 % in $Y_3Fe_5O_{12}$, which could in principle modulate the plasmonic-spin-pumping signal as the pumped spin current is proportional to $\Gamma^+\Gamma^-$ (see equation (13)). However, such anisotropy effects are irrelevant to the observed voltage signals for the following reasons. Firstly, the anisotropy effects do not explain the experimental fact that the observed voltage signal is an odd function of the external magnetic field (Fig. 4d in the main text); the possible modulation of the plasmonic spin pumping induced by the anisotropy of magneto-optical coefficients must be an even function of the magnetic field. Secondly, in the present system, the polarization of near-field photons is distributed randomly, and thus such anisotropy effects are smeared out in the net signal. These arguments justify our conclusion that the voltage signal in the Pt/$BiY_2Fe_5O_{12}$/Au-NP sample is not affected by the anisotropy of magneto-optical coefficients in $BiY_2Fe_5O_{12}$.

Supplementary References

1. Uchida, K. *et al.* Long-range spin Seebeck effect and acoustic spin pumping. *Nature Mater.* **10,** 737-741 (2011).
2. Uchida, K. *et al.* Acoustic spin pumping: direct generation of spin currents from sound waves in Pt/$Y_3Fe_5O_{12}$ hybrid structures. *J. Appl. Phys.* **111,** 053903 (2012).
3. Adachi, H., Uchida, K., Saitoh, E. & Maekawa, S. Theory of the spin Seebeck effect. *Rep. Progr. Phys.* **76,** 036501 (2013).
4. Adachi, H. & Maekawa, S. Linear-response theory of the longitudinal spin Seebeck effect. *J. Korean Phys. Soc.* **62,** 1753-1758 (2013).
5. Elliott, R. J. & Loudon, R. The possible observation of electronic Raman transitions in crystals. *Phys. Lett.* **3,** 189-191 (1963).
6. Shen, Y. R. & Bloembergen, N. Interaction between light waves and spin waves. *Phys. Rev.* **143,** 372-384 (1966).
7. Moriya, T. Theory of light scattering by magnetic crystals. *J. Phys. Soc. Jpn.* **23,** 490-500 (1967).
8. Fleury, P. A. & Loudon, R. Scattering of light by one- and two-magnon excitations. *Phys. Rev.* **166,** 514-530 (1968).
9. Bass, F. G. & Kaganov, M. I. Raman scattering of electromagnetic waves in ferromagnetic dielectrics. *Soviet Phys. JETP* **10,** 986-988 (1960).
10. Loudon, R. Theory of the temperature dependence of first-order light scattering by ordered spin systems. *J. Phys. C: Solid State Phys.* **3,** 872-890 (1970).
11. Wettling, W., Cottam, M. G. & Sandercock, J. R. The relation between one-magnon light scattering and the complex magneto-optic effects in YIG. *J. Phys. C: Solid State Phys.* **8,** 211-228 (1975).
12. Demokritov, S. O., Hillebrands, B. & Slavin, A. N. Brillouin light scattering studies of confined spin waves: linear and nonlinear confinement. *Phys. Rep.* **348,** 441-489 (2001).
